# Pain Assessment and Management in Pediatric Intensive Care Units Around the World, an International, Multicenter Study

**DOI:** 10.3389/fped.2021.746489

**Published:** 2021-10-28

**Authors:** Michelle Grunauer, Caley Mikesell, Gabriela Bustamante, Gissela Cobo, Sebastián Sánchez, Ana María Román, Andrea P. Icaza-Freire, Antonio W. D. Gavilanes, Nancy Ewen Wang, The PICU-MIC Research Group, Jorge López González

**Affiliations:** ^1^School of Medicine, Universidad San Francisco de Quito USFQ, Quito, Ecuador; ^2^Pediatric Intensive Care Unit, Hospital de los Valles, Quito, Ecuador; ^3^Department of Pediatrics, University Medical Center Maastricht, Maastricht, Netherlands; ^4^Emergency Medicine, Stanford University School of Medicine, Stanford, CA, United States

**Keywords:** pain, pediatric critical care units, pediatric palliative care, under resourced settings, pediatric

## Abstract

The adequate assessment and management of pain remains a challenging task in the Pediatric Intensive Care Unit (PICU). Our goal is to describe how pain is assessed and managed in PICUs around the world and to examine how human and material resources impact achievement of this goal. An international multicenter cross-sectional observational study was designed with the participation of 34 PICUs located in urban, suburban, and rural areas of 18 countries. We evaluated how PICUs around the world assessed and managed pain according to the Initiative for Pediatric Palliative Care recommendations, and how human and material resources impacted achievement of this goal. Data was collected for this study from 2016 to 2018 using questionnaires completed by medical doctors and nurses. In this paper, we focus on the indicators related to how pain is managed and assessed. The average achievement of the goal of pain relief across all centers was 72.2% (SD: 21.1). We found a statistically significant trend of more effective pain management scores, routine assessment, proper documentation, and involvement of pain management experts by increasing country income. While there are efforts being made worldwide to improve the knowledge in pain assessment and management, there is a lack of resources to do so appropriately in low-middle-income countries. There is a mismatch between the existing guidelines and policies, which are mainly designed in high income countries, and the resources available in lower resourced environments.

## Introduction

An inevitable consequence of a child's admission to the intensive care unit is the experience of pain, either because of the need for painful procedures or because of the disease itself. In this context, prevention of pain and pain management is fundamental (1). All critical care providers receive training in pain control and should apply it in an integrated model of care considering the principles related to palliative care: the active total care of the child's body, mind and spirit (2). In this context, Cicely Saunders, a founder of the discipline of Palliative Care, developed the concept of addressing “total pain” or the importance of addressing pain not only from a physical standpoint, but also from psychological, social, and spiritual aspects of life (3, 4). This approach to pain management has been demonstrated to positively affect the patient-family unit care, provide symptom control, and also improved survival among pediatric patients with life limiting and life-threatening conditions (1, 2). Nevertheless, a significant number of children still experience a lack of optimal pain management, which can lead to long and short-term psychological and physiological disturbances (5).

Despite the fact that these conversations and definitions are gaining prominence in both the scientific literature and in clinical settings, in 2008, the World Health Organization (WHO) estimated that, ~80% of the worldwide population has scant or no access to treatment for moderate to severe pain of various etiologies (1). The challenge for health care professionals is to find a way to assess and manage pain despite the fact that the sensation of pain is subjective and that there is poor use of standardized methods to evaluate pain in children (6). The gold-standard for diagnosing pain in the pediatric population is the use of self-reported scales. However, this method has limitations depending on the patient's age or acuity (5). For example, in very young children and in neonates, pain assessment includes scales that consider behavioral observations and physiological measures (7). As an alternative, Franck et al. (6) mentions that parents and non-professionals are more accurate than healthcare providers in identifying expressions and responses related to pain in children. Furthermore, there are multiple factors that affect pain perception including: anxiety, fear, stigma, comorbidities, and concern of separation from family, strange environments, and barriers in verbal communication (8).

Thus, the adequate assessment and management of pain remains a challenging task in Pediatric Intensive Care Units (PICUs). This can be attributable to complications in pain assessment and management in pediatric patients arising from a variable understanding of illness and death depending on the age of the child, as well as different stages of cognitive and emotional development (9, 10). Age-related differences in expressing pain also make assessment challenging (10). There are many barriers that practitioners confront in everyday practice, including access to validated tools to assess and treat pain, deficient practitioner training, a lack of pain experts, lack of time required to properly assess pain, and interruptions in the supply of pain medications (1, 11, 12).

Another aspect to consider while analyzing pain, is how racial bias can influence pain perception, defined as: an inequality in pain treatment between races despite showing similar levels of pain (13). Furthermore, social and cultural differences affect the way patients experience and exhibit pain. For instance, in some cultures, expression of emotions and acknowledgment of the pain is valued, whereas in others, stoicism is valued (14). Additionally, several studies have demonstrated that boys are rated as experiencing more pain than girls when undergoing the same medical procedures. Therefore, gender stereotypes, such as boys being more stoic than girls, also becomes a limitation (15).

The lack of proper assessment of pain leads to inadequate pain relief. Pain can limit the ability to perform daily activities of living. This can trigger psychosocial instability manifested as depression, anxiety, and a patient-family unit's inability to participate in work or studies (1, 4). Finally, spiritual pain as part of total pain is recognized as anger, hopelessness, and a sense of injustice (3).

In order to provide care to children living with life-threatening conditions, as well as their families with an integrated approach, the Initiative for Pediatric Palliative Care developed 6 quality domains including relief of pain (16). As part of an international multicenter cross-sectional study, we assessed how PICUs around the world assessed and managed pain in relation to the Initiative for Pediatric Palliative Care recommendations, and examine how human and material resources impact achievement of this goal.

## Materials and Methods

The international PICU-Model of Integrated Care (PICU-MIC) multicenter study identified institutions through medical societies, the Pediatric Acute Lung Injury and Sepsis Investigators Network, publication database searches and team contacts (17). An international multicenter cross-sectional observational study was designed with the participation of 34 PICUs located in urban, suburban and rural areas of 18 countries. Each institution designated a representative investigator who oversaw the study protocol and acquired Institutional Review Board (IRB) approval. Data collection took place from 2016 to 2018, and consisted of two questionnaires with multiple choice and open-ended questions completed by medical doctors and nurses directly caring for children from the 34 PICUs in the PICU-MIC network (18). Each PICU had a designated site coordinator who ensured that surveys were completed. Participants were encouraged to complete questionnaires on REDCap, (19) an encrypted, password-protected online platform. Respondents who could not use REDCap because of a lack of reliable internet sent de-identified responses *via* email. For each center, a two-week period was established to complete the requested questionnaires. The authors did not specifically take into account which caretakers were responsible for pain scoring and management. The first questionnaire collected data related to PICU infrastructure, technology, and provider ratios. The second questionnaire asked providers to answer questions related to providers' practices and center policies based on the Initiative for Pediatric Palliative Care's (IPPC) curriculum that describes domains, goals and indicators for the provision of pediatric palliative care. Each health provider that completed the questionnaire did so while considering the admitted child and the questions related to the domains applied in the care of each child. These questionnaires included 10 to 25 admitted children in each PICU with a 100% survey completion rate. De-identified data were collected using encrypted software (REDCap). This project received approval by the Universidad San Francisco de Quito's Ethics Committee for Research on Human Beings/IRB (2016-0911N) and by ethics committees at all sites [Clinical registry number: ISRCTN12556149 (DOI 10.1186/ISRCTN12556149)].

The IPPC curriculum domains include: (1) holistic care, (2) family support, (3) child-family unit involvement, (4) pain and symptom control, (5) continuity of care, and (6) grief and bereavement support. In this paper, we focus exclusively on the individual indicators related to how pain is managed and assessed. The pain domain assesses four items (a) Is pain evaluated? (b) What tools are used for pain evaluation? (c) Is pain level reported in the patient's chart? (d) Is pain assessment focused on a specific marker such as expressed, observed, physiological indicators, family reports or the child's ability to participate in activities of daily living? Questions regarding appropriate pain treatment and planning were also addressed: (a) Is there a dynamic therapeutic plan for the patient's pain with a wide range of pharmacologic and non-pharmacologic management strategies? (b) Are there specialists/experts involved in pain management? (c) Does the unit have policies regarding treatment of pain?

Each item had a possible response of “yes,” “no,” and “sometimes.” To analyze adherence to the pain domain of the Initiative for Pediatric Palliative Care curriculum, we constructed a partial score for each subcategory. A numeric value was assigned to each answer within each subcategory: “yes” = 1, “sometimes” = 0.5, and “no” = 0. Scores from all items were summed and a range from 0 to 100 was produced. A model using the mean and standard deviation (SD) of the results was created.

We grouped the centers by income level according to the World Bank definitions for low-middle-income countries, upper-middle-income countries, and high-income countries to be able to determine whether a country's financial stability alters the availability and/or quality of pain management between institutions. The mean of the results of pain assessment and management were juxtaposed with the World Bank income group. The evaluation of these dissimilarities was determined with the application of multilevel generalized linear models (GLM) with a Gaussian distribution modified by age of the child and gender, with clustering by center. While utilizing the high-income country group as reference, the World Bank income group was modeled categorically and ordinally while using the high-income country group as a reference group to determine the existence of a linear trend across income groups. Additionally, we examined if the patient or center characteristic were associated with Initiative for Pediatric Palliative Care-adherence scores using univariate and multivariable multilevel GLM using the center as a clustering variable. Age, race, gender, comorbidities, and shift length were all included in the adjusted model. For demographic information and other patient characteristics we included age, gender, race, length of stay (LOS), diagnosis, and history of comorbidities. In regard to the centers, we reported information on percent of daily bed use, beds per critical care nurse/doctor, health care provider shift lengths and frequency of pain assessment. We determined associations with univariate and multivariable multilevel GLM utilizing the center as a clustering variable. The statistical analysis was made with Stata v14.1.

We included concepts of content analysis and grounded theory as a part of a mixed-methods methodology to analyze the participants' open-ended answers. The responses were stratified by World Bank income level following the extraction and categorization of responses by question. Later, we classified answers by categories, removed duplicates, and condensed answers when feasible. Lastly, we analyzed participants' responses by World Bank country income level to associate data received in open-ended answers to results from our statistical analysis and literature review.

## Results

The PICU-MIC study included 34 PICUs from 18 countries: Asia (15), Latin America (7), North America (5), Europe (5), and Africa (2), to analyze the achievement of “relief of pain and other symptoms”. PICUs were classified according to their income: low and lower middle income (LIC/LMIC) (23·5%), upper middle income (UMIC) (44·1%) and high income (32·4%).

As shown in Table 1, the average achievement of the goal of pain relief across all centers was 72·2% (SD: 21·1). We found a statistically significant trend of increasing pain management scores by increasing country income: LICs/LMICs showed 62·6% (SD: 27·6), while UMICs 70·1% (SD: 20·0), and high-income countries showed 80·4% (SD: 13·8; *p*-value for trend: 0·03).

**Table 1:** Average scores for initiative for pediatric palliative care indicators of relief of pain and other symptoms (*each child living with a life-threatening condition receives effective pain and symptom management*) by World Bank income level (16).

**Relief of pain and other symptoms—quality indicators**	**Score^‡^**	**World bank income level**			
		**Low and lower middle income**	**Upper middle income**	**High income**	**All centers**
**Overall**	Mean (sd)	62.6 (27.6)	70.1 (20.0)	80.4 (13.8)	72.2 (21.1)
	p-trend^*^				***0.03***
Routine assessment	Mean (sd)	89.0 (26.2)	97.1 (13.4)	99.4 (7.6)	96.3 (15.9)
	p-trend^*^				***0.004***
Assessment documented	Mean (sd)	77.0 (38.5)	92.5 (21.3)	94.7 (17.2)	90.2 (25.4)
	p-trend^*^				***0.02***
Pain assessment focus:					
Expressed pain	Mean (sd)	84.0 (35.4)	85.5 (34.0)	80.7 (28.8)	83.5 (36.0)
	p-trend^*^				*0.50*
Observed pain	Mean (sd)	93.0 (23.6)	96.3 (14.8)	95.6 (20.2)	95.4 (18.7)
	p-trend^*^				*0.27*
Physiological indicators	Mean (sd)	84.0 (34.7)	90.1 (29.0)	82.2 (37.0)	86.1 (33.2)
	p-trend^*^				*0.79*
Family report	Mean (sd)	68.5 (36.0)	71.1 (43.3)	71.6 (43.7)	70.8 (42.0)
	p-trend^*^				*0.97*
Child's ability to perform daily activities	Mean (sd)	37.0 (45.8)	42.1 (47.5)	57.9 (48.3)	46.5 (48.1)
	p-trend^*^				*0.55*
Appropriate treatment plan	Mean (sd)	67.5 (42.9)	52.4 (38.3)	85.1 (32.5)	66.7 (40.0)
(pharmacologic and non-pharmacologic)	p-trend^*^				*0.20*
Pain management experts involved	Mean (sd)	55.5 (46.5)	57.3 (47.8)	73.7 (42.8)	62.6 (46.5)
	p-trend^*^				***0.04***
Guidelines and policies	Mean (sd)	33.5 (69.2)	69.2 (44.8)	68.7 (44.7)	61.8 (47.0)
	p-trend^*^				*0.40*

‡ *Scores range from 0–100%-points.*

* *p-trend, p-value for linear trend estimated using GLMs adjusted for child's age and gender, and using the center as a clustering variable.*

*sd, standard deviation.*

*Bold values represent statistically significant with a p-value of < 0.05*.

We also observed this overall trend of higher scores with increasing country income in several of the individual items assessed for relief of pain (Table 1). When routine assessment was analyzed the average score for centers in LICs/LMICs was 89·0% (SD: 26·2), compared to 97·1% (SD: 13·4) in UMICs and 99·4% (SD: 7·6) in high-income countries (*p*-value for trend: 0·004). Proper documentation of a pain assessment was achieved in 77·0% (SD: 38·5) among centers from LICs/LMICs, 92·5% (SD: 21·3) among centers from UMICs and 94·7% (SD: 17·2) for those in high-income countries (*p*-value for trend: 0·02). This data reveals increased frequency of routine pain assessment, as well as increased frequency of documented pain assessment in higher income countries in comparison to low-income countries.

We did not find differences across centers by country income in three other indicators, including the focus of pain assessment (i.e., expressed pain, observed pain, physiological indicators, family reports, child's ability for daily activities), having an appropriate treatment plan, and the existence of guidelines and policies for pain relief in each center (Table 1). However, we did find that centers in high income countries had higher scores for the involvement of pain management experts with a 73·7% (SD: 42·8), compared with 55·5% (SD: 46·5) in low-income/low-middle-income countries (*p*-value for trend: 0·04).

Table 2 represents the achievement of pain relief by sociodemographic characteristics of the patients and the centers included in the study. We did not find any statistically significant associations between patient and center characteristics and relief of pain in the fully adjusted model. However, in the univariate model, we found that teenagers (>11–18 years) had higher scores for pain relief compared to children of preschool age (>1–5 years). Similarly, there was a tendency identified in the univariate model of longer shifts having lower scores of pain relief compared with shifts of <8 h (*p*-value for trend 0·08).

**Table 2:** Associations between patient and center characteristics, and overall scores for initiative for pediatric palliative care indicators of relief of pain and other symptoms.

			**pain**		
**Patient Characteristics**	**N**	**%**	**Mean (sd)**	***p-value*** **^‡^**	***Adj. p-value***
**Characteristics**					
**Age**					
Newborn (0–1 m)	34	6.8	65.8 (27.4)	*0.95*	*0.50*
Infant (>1–12 m)	122	24.5	72.4 (19.8)	*0.60*	*0.97*
Preschool (>1–5 y)	150	30.1	70.4 (21.5)	*Ref*.	*Ref*.
School age (>5–11 y)	103	20.7	71.3 (19.9)	*0.25*	*0.35*
Teen (>11–18 y)	89	17.9	78.1 (19.6)	***0.03***	*0.12*
**Gender**					
M	285	57.2	71.9 (22.1)	*Ref*.	*Ref*.
F	213	42.8	72.5 (19.6)	*0.65*	*0.97*
**Race**					
White	173	34.7	74.3 (18.6)	*Ref*.	*Ref*.
Asian	111	22.3	78.3 (15.0)	*0.58*	*0.62*
Black	54	10.8	59.6 (30.4)	*0.19*	*0.22*
Indian	31	6.2	82.3 (11.3)	*0.96*	*0.81*
Mestiza	57	11.4	70.9 (27.3)	*0.61*	*0.64*
Middle-eastern	67	13.5	62.4 (16.6)	*0.44*	*0.99*
Other	4	0.8	82.0 (6.73)	*0.67*	*0.70*
**Days in PICU**					
<30 days	427	85.7	72.8 (21.1)	*Ref*.	*Ref*.
≥ 30 days	71	14.3	68.5 (20.9)	*0.22*	*0.50*
**Comorbidities**					
Single condition	133	26.7	70.7 (20.3)	*Ref*.	*Ref*.
Multiple comorbidities	365	73.3	72.7 (21.3)	*0.16*	*0.16*
**Main diagnosis**					
Acute	242	48.9	71.6 (20.8)	*Ref*.	*Ref*.
Chronic	253	51.1	72.7 (21.4)	*0.76*	*0.74*
**CENTER CHARACTERISTICS**					
**Percent daily bed use**					
<80%	139	30.2	76.4 (20.5)	*Ref*.	*Ref*.
≥80%	322	69.8	69.9 (21.0)	*0.88*	*0.89*
**Beds/critical care doctor**					
<2 beds per doctor	319	68.5	70.8 (20.1)	*Ref*.	*Ref*.
≥2 beds per doctor	147	31.5	72.0 (23.4)	*0.77*	*0.69*
**Beds/nurse**					
<2 beds per nurse	245	54.8	77.5 (19.3)	*Ref*.	*Ref*.
≥2 beds per nurse	202	45.2	65.1 (21.1)	*0.10*	*0.12*
**Shift length**					
<8 h	102	20.5	78.0 (12.4)	*Ref*.	*Ref*.
8 to 12 h	241	48.4	76.3 (19.4)	*0.99*	*0.60*
13 to 18 h	42	8.4	43.0 (19.1)	***0.01***	*0.04*
19 to 24 h	20	4.0	49.8 (31.4)	***0.05***	*0.32*
Multiple	93	18.7	72.9 (17.3)	*0.40*	*0.89*
*p-value for trend*				*0.08*	*0.39*

‡ *p-values were estimated using univariate and multivariable multilevel GLMs using center as a clustering variable. The adjusted model included all characteristics listed in the table.*

*Bold values represent statistically significant with a p-value of < 0.05*.

Finally, Table 3 shows that centers in countries of different incomes assess pain in PICU patients at different frequencies (Chi-square *p*-value < 0·001). In general, providers working in centers in high income countries reported that they assessed pain in the majority of their patients every 1–3 h (29%) or every 4–8 h (28%). Meanwhile, centers in upper-middle-income countries more frequently reported that they assessed pain “continuously,” “regularly” or “always” (37%), as opposed to assessing at a specific time interval. Centers in lower-middle-income countries did not show an identifiable response pattern with some assessing every 1–3 h (21%) or once/twice per day (25%).

**Table 3:** Frequency of pain assessment as reported by centers of different World Bank income levels.

Frequency of pain assessment^*^	**Low and lower middle income**		**Upper middle income**		**High income**		**All centers**	
	**n**	**%**	**n**	**%**	**n**	**%**	**n**	**%**
Every 1–3 h	21	21.0	17	7.5	50	29.2	88	17.7
Every 4–8 h	10	10.0	45	19.8	48	28.1	103	20.7
Once/twice per day	25	25.0	48	21.1	15	8.8	88	17.7
Continuously/regularly	18	18.0	85	37.4	16	9.4	119	23.9
At each clinical evaluation	0	0.0	8	3.5	21	12.3	29	5.8
As needed	4	4.0	7	3.1	6	3.5	17	3.4
Missing	22	22.0	17	7.5	15	8.8	54	10.8
Total	100	100	227	100	171	100	498	100

* *p-value < 0.001 using a Chi-square test*.

## Discussion

Average achievement of routine assessment and proper documentation for the relief of pain and other symptoms, were found to be inversely related to country income (Table 1). The involvement of pain management experts and the time dedicated to the assessment of pain were also associated with high-income countries (Tables 1, 3). These results are consistent with the literature. Matula et al. (20) discuss considerations regarding relief of pain in low-middle-income countries, including deficient knowledge, adverse beliefs in regard to a child's pain and its treatment, as well as specific cultural beliefs. There is also a strong influence and preference of traditional or alternative treatments in some of these regions, possibly leading to a delay in the pain assessment or to the refusal of medication (19). Furthermore, the lack of material and human resources in these settings result in a scarcity of pain medications, a shortage of appropriate pediatric formulations, and inadequate understanding of pediatric dosing which in turn can cause suboptimal pain relief (20). These issues pertain especially to rural areas due to a paucity of pain specialists, who tend to practice in major cities (19). Moreover, there are misunderstandings among health care providers working in lower-middle-income countries regarding the adverse effects of opioid analgesics, the validity of self-reported pain scales, as well as a lack of institutional policies and guidelines (20). In contrast, high income countries possess the resources to treat pain in pediatric populations with the help of specialists or other physicians with pain management training. They offer a wide arrange of services including medication, procedures, psychological and physical therapy, and alternative medicine (21).

There was not a statistically significant difference present by country income in the indicators of pain assessment focus, appropriate treatment plan, and existence of guidelines and policies. This finding could translate to the new efforts being made worldwide to improve the knowledge in pain assessment and management, but the lack of resources to do so appropriately in lower-middle-income countries. There is a mismatch between the existing guidelines and policies of palliative care and pain management, which are mainly designed in high income countries, and the resources available in lower resourced environments (21). Due to this, low-income countries should prioritize their focus on the development of multidisciplinary teams that could apply low-cost treatment plans and educate professionals and family members alike, when the cultural context requires it (21).

Finally, the univariate model showed a higher prevalence of pain relief in teenagers in comparison to children in the preschool age. For context, the assessment of pain can be approached in the pediatric population by three different methods: self-report scales, observed behavioral changes and measured physiological indicators. Unfortunately, the number of available methods diminish progressively as the age of the child decreases. Preschool children are not developmentally able to utilize self-report scales and require alternative techniques to assess their pain (22). However, these scales are the easiest to use for untrained professionals who do not have the proper knowledge in regard to pain assessment and management. Thus, hindering evaluation and reducing the possibility of pain relief. Furthermore, the behavioral tools applied to younger children can be affected by severity of disease, stage of development and in neonates, gestational age. Additionally, older infants and toddlers could deliberately change the nature and intensity of their responses in function of pain anticipation (22).

This study has some limitations. Our sample was not generalizable. Centers were diverse, located in countries with different income levels and in different parts of the world. However, our sample offered insight into areas often excluded from research as a consequence of geographic, linguistic or resource barriers. Furthermore, we were not able to differentiate centers by public, private or public-private institutions, nor urban, suburban or rural localities.

## Conclusions

Pain management remains a challenging task in the pediatric population, especially in the severely ill child. Furthermore, very young children and neonates have less available tools for the assessment of pain. Evidence suggests that the implementation of adequate pain assessment and treatment not only directly benefits the child by providing symptom control and quality of life, but also improves family and the health care professional's wellbeing. Our findings indicate that health care professionals already complete many palliative care tasks in PICUs around the world, independent of income. Despite this, there is an evident difference in fulfillment when World Bank income level is considered. Development, education, and barriers related to the implementation of evidence-based guidelines likely shaped Initiative for Pediatric Palliative Care pain scores. Moreover, there is a deficiency of material and human resources in countries with lower World Bank income levels, making it harder to implement the guidelines.

Understanding application of and adherence to pediatric palliative care guidelines can maximize the implementation effective interventions like the Initiative for Pediatric Palliative Care pain scores. Additionally, these recommendations should be adapted to each setting's available resources and inherent characteristics.

## Data Availability Statement

The original contributions presented in the study are included in the article/supplementary material, further inquiries can be directed to the corresponding author.

## Ethics Statement

The studies involving human participants were reviewed and approved by Universidad San Francisco de Quito's Ethics Committee for Research on Human Beings/IRB (2016-0911N) and by ethics committees at all sites [Clinical registry number: ISRCTN12556149 (DOI: 10.1186/ISRCTN12556149)]. Written informed consent for participation was not required for this study in accordance with the national legislation and the institutional requirements.

## Author Contributions

MG, CM, GB, and NW: substantial contributions to the conception or design of the work and the acquisition, analysis, or interpretation of data for the work. MG, CM, GB, GC, SS, AR, AI-F, AG, and NW: critical revision for important intellectual content and agreement to be accountable for all aspects of the work in ensuring that questions related to the accuracy or integrity of any part of the work are appropriately investigated and resolved. MG, CM, GB, GC, SS, AR, and NW: final approval of the version to be published. NW: critical revision for important intellectual content. GC, SS, and AR: literature review. PICU-MIC Research Group investigators: substantial contributions to the conception or design of the work and the data acquisition. All authors contributed to the article and approved the submitted version.

## Funding

This project was funded by Universidad San Francisco de Quito, Collaboration and Medical School's Grants.

## PICU-MIC Investigators

Jorge López González, Jesús López-Herce: Hospital General Universitario Gregorio Marañón-UCIP, Facultad de Medicina Universidad Complutense de Madrid, Madrid, Spain; Emanuele Rossetti, Chiusolo Fabrizio: Ospedale Pediatrico Bambino Gesù-PICU, Roma, Italy; Oliver Karam, Marie Saint-Faust: Geneva University Hospitals-HUG PICU, Geneva, Switzerland; Paolo Biban, Silvia Carlassara: Azienda Ospedaliera Universitaria Integrata Verona-PICU, Verona, Italy; Bettina von Dessauer, Nadia Ordenes: Hospital de Niños Roberto del Río Unidad de Paciente Crítico Pediátrico, Santiago, Chile; Fabiola Figueroa Urízar, Adriana Wegner A: Hospital Sótero Del Río-UCIP, Santiago, Chile; Michael Canarie, Yale-New Haven Children's Hospital's Pediatric Intensive Care Unit, New Haven, CT, United States, Kathryn Miller: University Hospitals Cleveland Medical Center-PICU, Cleveland, OH, United States. José Irazuzta; Nicolas Chiriboga: University of Florida COM-PICU, Jacksonville, FL, United States; Daniel Tawfik, Barbara Sourkes, Nancy Ewen Wang, Hursuong Vongsachang: Division of Pediatric Critical Care Medicine at Stanford University Medical Center-PICU, Stanford, CA, United States; Elizabeth W. Tucker, Nicole Shilkofski: The Johns Hopkins Children's Center PICU, Baltimore, MD, United States; 王文超 Wang, Wenchao RN, Zhang Yuxia RN: Pediatric Hospital of Fudan University-PICU—Fudan, China; Lucy Lum Chai See, Sister Priscilla: University Malaya Medical Center-PICU, Kuala Lumpur, Malaysia; Recep Tekin, Fesih Aktar: Dicel University Medical Hospital-PICU, Diyarbakir, Turkey; Duygu Sönmez Düzkaya, Istanbul University Faculty of Medicine PICU; Istanbul, Turkey; Oguz Dursun, Ebru Atike Ongun: Akdeniz University-Faculty of Medicine PICU, Antalya, Turkey; Resul Yilmaz, Gaziosmanpasa University School of Medicine Tokat- PICUTokat, Turkey; Dincer Yildizdas, Çukurova University, Faculty of Medicine; Balcali Hospital, PICU- Adana, Turkey; Hakan Tekgüç, Koru Hospital-PICU; Ankara, Turkey; Vitaliy Sazonov, Timur Tsoy: PICU of City Children Hospital; Astana, Kazakhstan; Vitaliy Sazonov, Askhat Saparov: PICU of National Research Center for Maternal and Child Health, Astana, Kazakhstan; Vitaliy Sazonov, Elizaveta Kalmbakh: PICU of Karaganda Regional Children's Hospital, Karaganda, Kazakhstan; Michelle Grunauer, (USFQ/HDLV); Ernesto Quiñones, Hospital de los Valles, Quito Ecuador; Luis Eguiguren, Universidad San Francisco de Quito, Quito, Ecuador; Killen Harold Briones Claudette, Centro Fisiológico-Respiratorio Briones-Claudett, Guayaquil, Ecuador; ICU Hospital General del IESS de Babahoyo, Babahoyo, Ecuador; Yaneth Tovilla, Unidad Pediátrica de Quemados de los Servicios de Salud del Estado de Puebla, Puebla, México; Sandra Tania Ventura Gómez, UCIP del Hospital Regional de Alta Especialidad de Ixtapaluca, Ixtapaluca, México; Silvio Fabio Torres, Pediatric Intensive Care Unit, Hospital Universitario Austral, Buenos Aires, Argentina; Paul Cobarrubias, Amang Rodriguez Memorial Medical Center-PICU, Manila, Philippines; Dmytro Dmytriiev, Vinnitsa National Medical University-PICU, Vinnitsa, Ukraine; Alejandro Martínez, Gustavo Guzaman, Rudy Sanabria: Hospital del Niño Manuel Ascencio Villarroel, Cochabamba-UTIP, Cochabamba, Bolivia; Ravikumar Krupanandan, Bala Ramachandran: PICU, Kanchi Kamakoti CHILDS Trust Hospital, Chennai, India; Nirmal Choraria, Jignesh Patel: PICU—Nirmal Hospital, Ltd., Surat, India; Puneet A Pooni, Karambir Singh Gill: Dayanand Medical College & Hospital-PICU, Punjab, India; John Adabie Appiah, Komfo Anokye: Teaching Hospital—PICU, Kumasi, Ghana; Tigist Bacha Heye, Rahel Argaw, Asrat Demtse, Israel Abebe Admasu: Addis Ababa University, College of Health Sciences-PICU, Addis Ababa, Ethiopia.

## Conflict of Interest

The authors declare that the research was conducted in the absence of any commercial or financial relationships that could be construed as a potential conflict of interest.

## Publisher's Note

All claims expressed in this article are solely those of the authors and do not necessarily represent those of their affiliated organizations, or those of the publisher, the editors and the reviewers. Any product that may be evaluated in this article, or claim that may be made by its manufacturer, is not guaranteed or endorsed by the publisher.
